# Exploring relationship of poor sleeping habits with prenatal stress among pregnant women in Pakistan: a cross-sectional study

**DOI:** 10.1186/s13104-024-06756-1

**Published:** 2024-04-19

**Authors:** Ahmed Waqas, Irfan Siddique, Mehroz Ahsen, Muhammad Zubair, Mehak Naeem, Aamir Raoof Memon, Sadiq Naveed

**Affiliations:** 1 Department of Primary Care & Mental Health, Institute of Population Health, University of Liverpool, Liverpool, UK; 2 Children Hospital and Institute of Child Health, Faisalabad, Pakistan; 3 Al-Shifa Hospital, Pattoki, Pakistan; 4 FMH College of Medicine and Dentistry, Lahore, Pakistan; 5 Institute for Health and Sport (IHeS), Victoria University, Melbourne, Australia; 6 Psychiatry Program Director, Eastern Connecticut Health Network, Manchester, CT USA

**Keywords:** PSQI, Sleep, Stress, Pregnancy, Pakistan, Exercise, Unplanned pregnancy

## Abstract

**Objective:**

Pregnancy is a complex phenomenon accompanied by biological, physiological and psychosocial changes for a mother. It is also regarded as a stressful life event where a woman’s role, identity and interpersonal relationships are restructured. The present study from Pakistan explores the association of sleep quality and poor sleeping habits with prenatal stress using Pittsburgh Sleep quality Index.

**Results:**

There were a total of 516 women (mean age = 29.82 years), with more than half reporting poor sleep quality. Ethnically, a majority (395, 76.6%) were natives of the Punjab province while rest were non-natives. A high percentage of respondents reported poor subjective sleep quality (22.1%), sleep latency (44.1%), habitual sleep efficiency (27.5%), sleep disturbance (30.1%), use of medications (7.1%) and daytime dysfunction (29.5%). According to logistic regression analysis, respondents with poor sleep quality were 2.24 (95% CI = 1.55–3.22, *P* < 0.001) times more likely to have high stress levels (*P* < 0.001).

**Supplementary Information:**

The online version contains supplementary material available at 10.1186/s13104-024-06756-1.

## Introduction

Pregnancy is a complex phenomenon accompanied by biological, physiological, and psychosocial changes for a mother [1–3]. It is also regarded as a stressful life event where a woman’s role, identity and interpersonal relationships are restructured [4]. Moreover, as an anticipatory response to being a mother, conflicting emotions and mental states are also experienced by women during pregnancy [4, 5].

Sleep disturbances are the most widespread problems experienced by pregnant women, with their incidence rate ranging between 18% and 61% [1, 5]. A recent study conducted in Taiwan screened as many as 76% pregnant women for poor sleep quality [6]. Poor sleep quality during pregnancy has been associated with adverse maternal and neonatal outcomes including daily dysfunction, increased perception of pain and discomfort during labor, psychological distress, fatigue, gestational diabetes, depression, low birth weight, preterm birth, lengthy labor, and intrauterine growth retardation [5, 7, 8].

Prenatal stress has been reported as a significant predictor of sleep disturbances during pregnancy [2, 5, 9, 10]. The evidence for prenatal stress contributing to insomnia has also been demonstrated in animal studies [1, 11]. However, robust prospective studies rather show a bidirectional relationship between chronic sleep problems and stress [12–14]. Both biological and psychological stress during pregnancy contribute to altered sleep quality in 66–94% of the pregnant women [8], affecting both the subjectively reported and objectively measured sleep quality [5].

Sleep disorders are prevalent in lower- and middle-income countries (LMICs) and significantly contribute to morbidity and mortality resulting from complications during pregnancy and childbirth [15]. To the best of our knowledge, there are a few studies from Pakistan that have attempted to explore the prevalence of prenatal sleep problems and its risk factors. Moreover, research on the relationship between sleep and prenatal stress from other parts of the world is also limited [12, 16], thus, warranting immediate public health attention.

Therefore, the present study was conducted to delineate the prevalence of sleep problems among prenatal women in Pakistan. It also aims to explore factors associated with sleep problems among prenatal women, focusing on individual-level demographic and clinical variables such as trimester of pregnancy, household income, education, and occupation.

## Materials and methods

### Study setting

This cross-sectional study was conducted from November 2016 to March 2017, at obstetrics and gynecology departments of District Headquarter Hospital and Children Hospital in district Faisalabad and a rural health center in Charanwala Village, district Mandi Bahauddin (Pakistan Bureau of Statistics). These hospitals in Faisalabad are major teaching sites for undergraduate and postgraduate medical students, serving an urban population exceeding 4 million, along with adjoining rural areas. The village of Charanwala comprises 162 households and 1036 individuals (Pakistan Bureau of Statistics). These three study sites consequently offered a diverse study sample that was representative of both the urban and rural inhabitants in the Punjab region.

### Sampling procedures

A non-probability convenience sampling procedure was implemented in our study, where every fifth patient at the obstetrics and gynecology departments of the chosen hospitals was interviewed. This method was adopted due to the limited number of interviewers available - a team consisting of six physicians, nurses, and medical students. Our personnel constraints necessitated this approach, as random sampling was not feasible, ensuring efficiency in data collection despite the resource limitations. Participation in this study was voluntary and all participants and their legal guardians signed the informed consent form.

We invited all adult women (aged 18 and older) to participate in the study who visited the selected hospitals for obstetric care. We adopted an inclusive approach, ensuring that no woman was excluded based on age, background, religion, or ethnicity. However, our criteria did necessitate the exclusion of women who were not proficient in Punjabi, Urdu, or Saraiki. Additionally, we excluded women presenting with significant medical comorbidities that required inpatient hospitalization, such as hypertensive pregnancy disorders, and severe psychiatric conditions including psychosis and intellectual disabilities. Women requiring such inpatient care were directed to appropriate specialist services for more specialized attention.

### Ethics

Ethical approval for this study was obtained from the ethical review committee at the FMH College of Medicine and Dentistry, Lahore, Pakistan (Ethical review certificate number: FMH-06-2017-IRB-261-M). All methods were performed in accordance with the ethical principles outlined in the Declaration of Helsinki for medical research involving human subjects. This manuscript follows the STROBE guidelines for reporting of cross-sectional studies. Details on study methods are provided elsewhere [17].

### Instruments

All respondents were interviewed by the research team, using the preformed questionnaire consisting of four parts: (1) variables related to demographic characteristics and obstetric history; (2) a 4- item perceived stress scale (PSS-4) and (3) an Urdu version of Pittsburgh Sleep Quality Index (PSQI-U). Results for variables related to gender and health disparity have been reported in another study [17]. Before conducting the project, twenty pregnant women were recruited and interviewed to ascertain the comprehensibility of the questionnaire.

A 4-item Perceived Stress Scale (PSS-4), exhibiting good internal consistency in the present study sample (Cronbach’s alpha = 0.793), was employed to evaluate stress among respondents. As indicated in literature, stress levels were dichotomized into low and high, by combining upper and lower two quartiles [18, 19].

The Pittsburg Sleep Quality Index (PSQI) is a 19-item self-rating questionnaire designed to evaluate sleep quality and disturbances during the last month. It is a reliable and valid tool for use in pregnant women [20]. It consists of seven components namely (i) subjective sleep quality, (ii) sleep latency, (iii) sleep duration, (iv) habitual sleep efficiency, (v) sleep disturbance, (vi) use of sleeping medication and (vii) daytime dysfunction [20]. The PSQI item scores range from 0 to 3 which are then summed to obtain a global score that ranges from 0 to 21. Higher scores on PSQI indicate poor sleep quality. Participants with global PSQI score > 5 are considered poor sleepers (89.6% sensitivity and 86.5% specificity) [20]. We used Urdu-version of PSQI for this study which has a good linguistic interchangeability and test-retest reliability [21].

### Statistical analysis

For prevalence estimation of sleep problems, a minimum sample size of 377 was deemed appropriate based on 95% confidence level, 5% margin of error and 50% response distribution. G-Power was used to estimate minimum sample size requirements for inferential statistics. To achieve a statistical power of 80% for weak to moderate strengths of associations, a minimum sample size of 343 was required. Moreover, following rules of thumb for sample size estimation for logistic regression analyses, the most conservative estimates recommend 20 participants per predictor, thus, indicating a sample size of 240 to be appropriate [22]. However, we approached 550 study women, as available resources permitted us to perform oversampling in the study. This decision was made to enhance the robustness of our findings and to potentially compensate for any unforeseen non-response, which, albeit estimated at around 10%, could vary in practice.

All data were analyzed in SPSS v.20 (IBM, Illinois, USA). Categorical variables were presented as frequencies and quantitative as mean (SD). Correlation between quantitative variables was assessed with Pearson correlation and Point-biserial correlation was used to assess association between binary variables and quantitative variables. Predictors of stress levels were identified by conducting a binary logistic regression analysis, which included demographic, clinical, and obstetric variables. Finally, multiple regression analysis and Sobel test were used to assess mediation effects of stress levels on association of unplanned pregnancy with poor sleep quality. *P*-value < 0.05 was considered significant.

## Results

### Characteristics of respondents

From the 550 individuals approached, 516 agreed to be part of the study, resulting in a participation rate of 93.82%. The mean age of respondents was 29.82 (6.50) years. Among socioeconomic groups, most respondents belonged to the lower and upper-middle class totaling 364 (70.54%), low class 135 (26.2%), and Upper class 17 (3.3%). In terms of educational qualifications, the majority of respondents had education ranging from primary to high school, totaling 260 (50.39%), and college 122 (23.64%). A total of 134 (26%) of the respondents could not read and write. There were 260 (50.4%) respondents from rural areas, 185 (35.9%) from semi-urban and 71 (13.8%) from urban areas. Most of the women were housewives 426 (82.6%) while rest were employed. Ethnically, a majority 395 (76.6%) were natives of the Punjab province while rest were non-natives. Their mean score on perceived stress scale was 7.55 (3.43). More than half of the study participants had a planned pregnancy, numbering 303 (58.70%), compared to those with an unplanned pregnancy.

### Pittsburgh sleep quality index

More than half of the respondents reported poor sleep quality. A substantial percentage of respondents reported poor subjective sleep latency, at 44.1%, sleep disturbance (30.1%), daytime dysfunction (29.5%), poor habitual sleep efficiency (27.5%) and sleep quality (22.1%) and use of medications (7.1%). Detailed results are given in Table 1. Association between different domains of sleep quality and global PSQI scores are given in supplementary Table 1. Poor PSQI scores were significantly associated with all domains of the PSQI scale.

**Table 1:** Domains of sleep quality

Variable	Subcategory			
	Better	Good	Bad	Worse
Subjective sleep quality	50 (9.70%)	352 (68.20%)	97 (18.80%)	17 (3.30%)
Sleep latency	128 (24.90%)	161 (31.10%)	140 (27.20%)	87 (16.90%)
Sleep duration	308 (59.70%)	136 (26.40%)	58 (11.20%)	14 (2.70%)
Habitual sleep efficiency	301 (58.30%)	73 (14.10%)	67 (14.50%)	75 (14.50%)
Sleep disturbance	6 (1.20%)	347 (68.70%)	152 (30.10%)	0
Use of medication	427 (82.80%)	52 (10.10%)	25 (4.80%)	12 (2.30%)
Daytime dysfunction	114 (22.10%)	250 (48.40%)	132 (25.60%)	20 (3.90%)
PSQI global		226 (44.80%)		290 (55.20%)

### Correlates and predictors of sleep quality

Poor sleep quality was not significantly associated with background (F = 0.074, *P* > 0.05), trimester (F = 1.361, *P* > 0.05), occupation (F = 1.57, *P* > 0.05), household income (*r*=-0.07, *P* > 0.05), and education levels (*r* = 0.02, *P* > 0.05). However, sleep quality was significantly worse in cases of unplanned pregnancy (*r* = 0.15, *P* = 0.001)and among women not consuming vitamin supplements during pregnancy (r_pb_= 0.20, *P* < 0.001).

### Domains of sleep quality as a predictor of stress

Their mean score on perceived stress scale was 7.55 (3.43). A total of 218 (42.2%) women reported high stress levels. According to unadjusted logistic regression analysis, respondents with poor sleep quality were 2.24 (95% CI = 1.55–3.22, *P* < 0.001) times more likely to have high stress levels (*P* < 0.001).

A logistic regression analysis using the backward method revealed a statistically significant model (*P* < 0.001). According to it, respondents with poor education levels and those experiencing poor sleep latency and poor sleep duration were more likely to develop high stress levels. And those experiencing sleep disturbance were less likely to report high stress levels. Compared with third trimester, stress levels were higher in second trimester. Detailed results are given in Table 2. Results for logistic regression analysis are given in supplementary Table 2.

**Table 2:** Backward logistic regression analysis delineating predictors of stress among pregnant women

Variables	Subcategories	B	S.E.	*P*-value	Odds ratio (OR)	95% C.I. for OR	
						Lower	Upper
Trimester	Third	1		0.032			
	First	0.366	0.270	0.175	1.442	0.850	2.446
	Second	0.605	0.230	0.009	1.831	1.166	2.877
Household income		0.256	0.150	0.087	1.292	0.964	1.733
Education levels		-0.187	0.089	0.035	0.829	0.697	0.987
Sleep latency	Good				1		
	Worse	0.785	0.194	< 0.001	2.192	1.500	3.203
Sleep duration	Good				1		
	Worse	0.982	0.288	0.001	2.671	1.519	4.697
Sleep disturbance	Good				1		
	Worse	-1.097	0.225	< 0.001	0.334	0.215	0.519
Constant		− 0.913	0.342	0.008	0.401		

### Unplanned pregnancy, stress and sleep quality

Stress exerted significant mediation effects when the status of pregnancy was explored as a predictor of sleep quality (Sobel test statistic = 3.346, SE = 0.002, *p* < 0.001). Higher stress levels exacerbated the effect of unplanned pregnancy on poor sleep quality.

## Discussion

The present study reports a high burden of poor sleep quality and psychological stress among Pakistani pregnant women. Sleep quality was worse among women with unplanned pregnancies and those not taking supplements. Higher levels of psychological stress were associated with overall poor sleep quality, worsening sleep latency and duration of sleep.

This high prevalence of sleep problems is corroborated by previous studies on sleep disorders during pregnancy. For instance, a cross-cultural study on pregnant women across 14 countries involving a large cohort of Caucasian and Asian mothers reported poor sleep in 55% women with the minimum (43%) in China and the maximum (78%) in Japan [23]. Insufficient sleep and interrupted sleep are common during the late stages of pregnancy [4, 24, 25].

According to our analysis, no trimester-specific changes in sleep quality were reported among our respondents. This is in contrast to previous studies that have reported trimester-specific changes in sleep quality among pregnant women [1]. According to Reichner, sleep disturbances range from 13%, 19% and 66% in the first, second and third trimester respectively [7]. These trimesters specific disposition to insomnia, sleep deficiency and its severity corresponds to the physiological and psychological changes that occur during different trimesters. Additionally, due to the secretion of oxytocin, a wake-promoting hormone, sleep disturbances generally worsen right before labor [7].

In this study, a high percentage of respondents reported poor subjective sleep quality (22.1%), sleep latency (44.1%), habitual sleep efficiency (27.5%), sleep disturbance (30.1%), use of medications (7.1%) and daytime dysfunction (29.5%). Mindell and colleagues report poor sleep quality, insufficient nighttime sleep, significant daytime sleepiness and daytime naps in a high percentage of pregnant women [23]. Surprisingly, night time awakening was reported in almost all women (100%) in this study [26]. Similarly, reduced total time of sleep during pregnancy has also been reported in previous studies [5, 7, 26].

Our study reports that a high prevalence of stress was significantly associated with poor sleep quality, sleep latency, and sleep duration among the respondents. This relationship has been well explored in the literature, among various cultures and regions [27]. It can be explained using both psychological and neuro-endocrinological approaches. For instance, according to a cognitive model of insomnia, severe sleep problems during pregnancy may be found among women with a tendency towards high anxiety [17, 28]. Similarly, it can also be due to above mentioned endocrinological changes that accompany pregnancy, notably, high cortisol levels and altered melatonin and cortisol ratio as well as progesterone and its soporific effects [4, 29].

Interestingly, our study reports a negative association between stress levels and the experience of sleep disturbances. However, this finding might be spurious, with inferences regarding causality limited by the cross-sectional design of our study. It may also be due to the fact that this subdomain of PSQI does not take account of all the pregnancy specific sleep disturbances such as fetal movements [20].

### Limitations

The results of this study should be interpreted with caution. Firstly, due to its cross-sectional design, this study’s ability to determine the causal relationship between poor sleep habits and prenatal stress is limited. Longitudinal and prospective studies are needed to establish a causal relationship between poor sleeping habits and prenatal stress. Secondly, the reliance on subjective measures to assess sleep quality and prenatal stress may limit the reliability of this study’s results. Thirdly, due to resource limitations, we were unable to ensure random sampling procedures. Lastly, due to limitation of availability of research personnel, we could only select three centers for data collection. It is recommended that future investigators perform data collection procedures in the community or approach more medical centers to recruit a representative study sample.

Further research is needed that adopts multiple methodologies for the assessment of sleep and stress during pregnancy. We also recommend that anthropometric measurements of pregnant women should be noted in future studies.

### Electronic supplementary material

Below is the link to the electronic supplementary material.

Supplementary Material 1

## Data Availability

A confidentiality agreement with participants prevents us from sharing the data, therefore, dataset cannot be shared.
