# Supplementary material for: Exploring relationship of poor sleeping habits with prenatal stress among pregnant women in Pakistan: a cross-sectional study
Source: BMC Res Notes. 2024 Apr 19;17:110. doi: 10.1186/s13104-024-06756-1 (PMC11031876; doi:10.1186/s13104-024-06756-1)
Supplement: Supplementary file 1 — Supplementary Material 1 [file 13104_2024_6756_MOESM1_ESM.docx]

**Supplementary table 1: Association between domains of sleep quality and PSQI scores**

| **Overall sleep quality** | **Domains** | **Correlation coefficient** |
| --- | --- | --- |
| PSQI scores | Subjective sleep quality | .508^**^ |
|  | Sleep latency | .610^**^ |
|  | Sleep duration | .625^**^ |
|  | Habitual sleep efficiency | .607^**^ |
|  | Sleep disturbance | .362^**^ |
|  | Use of medication | .359^**^ |
|  | Daytime dysfunction | .422^**^ |

****denotes p < 0.001**

**Supplementary Table 2: Logistic Regression Analysis indicating association of sleep quality with stress (n=516)**

| **Variable** | **Subcategory** | **B** | **Std. error** | **P-value** | **Odds Ratio (OR)** | **95% C.I. for OR** | |
| --- | --- | --- | --- | --- | --- | --- | --- |
|  |  |  |  |  |  | **Lower** | **Upper** |
| Age |  | 0.014 | 0.016 | 0.392 | 1.014 | 0.983 | 1.046 |
| Ethnicity | Others |  |  |  | 1 |  |  |
|  | Punjabi | 0.430 | 0.263 | 0.102 | 1.538 | 0.918 | 2.576 |
| Trimester of pregnancy | Third |  |  | 0.027 | 1 |  |  |
|  | First | 0.397 | 0.280 | 0.156 | 1.488 | 0.859 | 2.576 |
|  | Second | 0.630 | 0.235 | 0.007 | 1.877 | 1.185 | 2.974 |
| Household income |  | 0.257 | 0.152 | 0.091 | 1.293 | 0.960 | 1.741 |
| Education levels |  | -0.125 | 0.095 | 0.189 | 0.882 | 0.732 | 1.063 |
| Subjective sleep quality | Good |  |  |  | 1 |  |  |
|  | Worse | 0.290 | 0.273 | 0.289 | 1.336 | 0.782 | 2.281 |
| Sleep latency | Good |  |  |  |  |  |  |
|  | Worse | 0.720 | 0.197 | <0.001 | 2.055 | 1.396 | 3.025 |
| Sleep duration | Good |  |  |  |  |  |  |
|  | Worse | 0.741 | 0.332 | 0.026 | 2.098 | 1.094 | 4.026 |
| Habitual sleep efficiency | Good |  |  |  |  |  |  |
|  | Worse | 0.289 | 0.252 | 0.252 | 1.335 | 0.815 | 2.187 |
| Sleep disturbance | Good |  |  |  |  |  |  |
|  | Worse | -1.142 | 0.252 | <0.001 | 0.319 | 0.195 | 0.523 |
| Use of medication | Good |  |  |  |  |  |  |
|  | Worse | 0.173 | 0.395 | 0.662 | 1.188 | 0.548 | 2.578 |
| Daytime dysfunction | Good |  |  |  |  |  |  |
|  | Worse | 0.059 | 0.230 | 0.796 | 1.061 | 0.676 | 1.665 |
| Constant |  | -1.928 | 0.694 | 0.005 | 0.145 |  |  |

Hosmer & Lemeshow Test P-value= 0.54, Chi square 7.672. Cox & Snell R^2^= 13.3%, Nagelkerke R^2^=17.9%, Significance of Model: Chi Square= 79.91, df 13, P < 0.001
